# Efficacy of high doses of oral penicillin versus amoxicillin in the treatment of adults with non-severe pneumonia attended in the community: study protocol for a randomised controlled trial

**DOI:** 10.1186/1471-2296-14-50

**Published:** 2013-04-17

**Authors:** Carl Llor, Javier Arranz, Rosa Morros, Anna García-Sangenís, Helena Pera, Joan Llobera, Mireia Guillén-Solà, Eugenia Carandell, Jesús Ortega, Silvia Hernández, Marc Miravitlles

**Affiliations:** 1 Primary Healthcare Centre Jaume I, University Rovira i Virgili, c. Felip Pedrell, 45-47, 43005, Tarragona, Spain; 2 Primary Healthcare Centre Arquitecte Bennàssar, Mallorca, IBSALUT, Spain; 3 Institut Universitari d’Investigació en Atenció Primària Jordi Gol, Barcelona, Spain; 4 Primary Healthcare Research Unit, Mallorca, Spain; 5 Primary Healthcare Centre Son Pisà, Mallorca, Spain; 6 Primary Healthcare Centre Rincón de Soto, Logroño, Spain; 7 Primary Healthcare Centre Jaume I, Tarragona, Spain; 8 Pneumology Department, Hospital Universitari Vall d’Hebron, Barcelona, Spain

**Keywords:** Community-acquired pneumonia, Penicillin, Amoxicillin, Adult

## Abstract

**Background:**

*Streptococcus pneumoniae* is the bacterial agent which most frequently causes pneumonia. In some Scandinavian countries, this infection is treated with penicillin V since the resistances of pneumococci to this antibiotic are low. Four reasons justify the undertaking of this study; firstly, the cut-off points which determine whether a pneumococcus is susceptible or resistant to penicillin have changed in 2008 and according to some studies published recently the pneumococcal resistances to penicillin in Spain have fallen drastically, with only 0.9% of the strains being resistant to oral penicillin (minimum inhibitory concentration>2 μg/ml); secondly, there is no correlation between pneumococcal infection by a strain resistant to penicillin and therapeutic failure in pneumonia; thirdly, the use of narrow-spectrum antibiotics is urgently needed because of the dearth of new antimicrobials and the link observed between consumption of broad-spectrum antibiotics and emergence and spread of antibacterial resistance; and fourthly, no clinical study comparing amoxicillin and penicillin V in pneumonia in adults has been published. Our aim is to determine whether high-dose penicillin V is as effective as high-dose amoxicillin for the treatment of uncomplicated community-acquired pneumonia.

**Methods:**

We will perform a parallel group, randomised, double-blind, trial in primary healthcare centres in Spain. Patients aged 18 to 65 without significant associated comorbidity attending the physician with signs and symptoms of lower respiratory tract infection and radiological confirmation of the diagnosis of pneumonia will be randomly assigned to either penicillin V 1.6 million units thrice-daily during 10 days or amoxicillin 1,000 mg thrice-daily during 10 days. The main outcome will be clinical cure at 14 days, defined as absence of fever, resolution or improvement of cough, improvement of general wellbeing and resolution or reduction of crackles indicating that no other antimicrobial treatment will be necessary. Any clinical result other than the anterior will be considered as treatment failure. A total of 210 patients will be recruited to detect a non-inferiority margin of 15% between the two treatments with a minimum power of 80% considering an alpha error of 2.5% for a unilateral hypothesis and maximum possible losses of 15%.

**Discussion:**

This pragmatic trial addresses the long-standing hypothesis that the administration of high doses of a narrow-spectrum antibiotic (penicillin V) in patients with non-severe pneumonia attended in the community is not less effective than high doses of amoxicillin (treatment currently recommended) in patients under the age of 65 years.

**Trial registration:**

EudraCT number 2012-003511-63.

## Background

*Streptococcus pneumoniae* is the bacterial agent which most frequently causes respiratory tract infections. Indeed, it is the most common bacteria in acute sinusitis, acute otitis media and community-acquired pneumonia. In some European countries, mainly those located in Northern Europe, pneumonia is treated with penicillin V since the resistances of these pneumococci to this antibiotic are low [1]. In the last years a phenomenon of greater community prescription of broad-spectrum antibiotics has been taking place in many countries, with amoxicillin-clavulanate and amoxicillin being the antibiotics most frequently prescribed by family practitioners in Spain [2,3]. Antimicrobial resistance may be a cause of therapeutic failure. It is identified by measuring the minimum inhibitory concentration (MIC) of the antimicrobial agent able to inhibit the growth of the microorganism. The cut-off points are MICs which define the infections as susceptible (treatable), with intermediate resistance (possibly treatable with higher doses) and resistant (not treatable). After re-evaluation taking new clinical studies into account, in January 2008 the *Clinical and Laboratory Standards Institute* published new cut-offs for penicillin [4]. These new cut-off points were determined on the basis of a series of criteria: first, by the pharmacokinetic and pharmacodynamic properties of the antimicrobial agents and second, by the data published relating MICs to the clinical outcome of the patients with pneumococcal infections. With the previous criteria a pneumococcus was considered to be susceptible with an MIC<0.06 μg/ml, to have intermediate resistance at between 0.12 and 2 μg/ml and to be resistant when the MIC was >2 μg/ml. At present, the new cut-off points for patients with meningitis receiving endovenous penicillin are <2, 4 and >8 μg/ml, respectively, unifying penicillin and amoxicillin. According to the results of the SAUCE 4 study published in 2010, the pneumococcal resistances to penicillin in Spain have fallen drastically, with only 0.9% of the strains being resistant to oral penicillin (MIC>2 μg/ml) [5]. In view of these results pneumonia could be treated with penicillin in Spain. In US the American Society of Infectious Diseases also recommends that patients with pneumonia be treated with penicillin if the causative strain of the infection is susceptible to this antibiotic [6].

### Study justification

Four reasons justify the undertaking of this study and these are: a) as mentioned above the cut-off points which determine whether a pneumococcus is susceptible or resistant to penicillin have changed; b) there is no correlation between pneumococcal infection by a strain resistant to penicillin and therapeutic failure in pneumonia. Treatment failure has been described in pneumonias treated with macrolides and fluoroquinolones. Curiously however, in contrast with the latter, in a review of clinical failures due to antimicrobial resistance no cases of therapeutic failure were reported with β-lactams prescribed at adequate doses [7]. It is important to know that a large proportion of patients die because of the severity of their disease and the associated comorbidity but not due to failure of the antibiotic. Thus, the study by Pallarés *et al.* has provided evidence that the risk of death by pneumococcal pneumonia treated with bencilpenicillin or ampicillin was similar regardless of whether the patients were infected with germs resistant or not to penicillin [8]. In other studies evaluating the effectiveness of penicillin in monotherapy for the treatment of non-meningeal pneumococcal infections within the first 48 hours in adults, an increase in mortality was not observed when the infection was caused by a strain with an MIC<2 μg/ml [9]. c) It is urgent to return to the use of reduced spectrum antibiotics. The increase in the use of broad-spectrum antibacterial agents has been accompanied by an increase in the rates of resistances of the microorganisms [10]. Moreover, the incidence of infection by *Clostridium difficile* is rising in parallel with an increase in the prescription of wide spectrum antibiotics [11]. d) No clinical study comparing amoxicillin and penicillin V in pneumonia in adults has been published. Amoxicillin is the first-choice option antibiotic for non-severe community-acquired pneumonia recommended by the Spanish Society of Family Medicine [12,13], and also endorsed by other scientific societies, such as the British Thoracic Society [14], and the European Respiratory Society/European Society for Clinical Microbiology and Infectious Diseases [15]. Although some clinical trials have compared amoxicillin with penicillin, these were performed in children and with parenteral doses [16-19].

## Methods

### Study hypothesis and objectives

The main hypothesis of this study is that the administration of high doses of penicillin V in patients with non-severe pneumonia attended in the community is not less effective than high doses of amoxicillin (currently recommended treatment) in patients under the age of 65 years without comorbidities. The main objective of this phase IV randomised, multicentre, clinical, double-blind, parallel, clinical trial is to evaluate the efficacy of phenoxymethylpenicillin (penicillin V) in the antibiotic treatment of non-severe pneumonia attended in the community compared with amoxicillin, considering the rate of clinical cure at 2 weeks. Secondary objectives are to assess the rate of radiological cure at 4 weeks in the patients treated with the two antibiotics, evaluate the rate of clinical cure at 4 weeks in both treatment groups, and evaluate the presence of adverse effects in the patients treated with the two antibiotics.

### Patient selection

Patients from 18 to 65 years of age without significant associated comorbidity attending the primary healthcare centres or emergency departments with signs and symptoms of lower respiratory tract infection and radiological confirmation of the diagnosis of pneumonia will be recruited in this clinical trial. Exclusion criteria will be subjects under 18 and over 65 years of age, severe impairment of signs (impairment of consciousness, respiratory rate > 30 breaths/minute, heart rate > 125 beats/minute, systolic blood pressure < 90 mm Hg, diastolic blood pressure < 60 mm Hg, temperature > 40°C, oxygen saturation < 92%), hypersensitivity to β-lactams, important alteration on chest X-ray, such as alveolar infiltrate in more than one lobe or bilateral, pleural effusion and/or pulmonary cavitation, problems to comply with treatment at home – sociopathy or psychiatric problems, drug and alcohol addiction, or within an inadequate family setting –, lack of tolerance to oral treatment, such as the presence of nausea and vomiting, gastrectomy, postsurgery and/or diarrhoea, significant comorbidity including bronchial asthma, renal failure, hepatic cirrhosis, heart failure, chronic obstructive pulmonary disease, ischaemic heart disease, stroke and/or type 1 diabetes mellitus, immunosuppression – chronic HIV infection, transplantation, neutropenic, or patients receiving immunosuppressive drugs or corticosteroids –, terminal disease, pregnancy or lactation, hospitalisation in the last month, consumption of antibiotics in the last two weeks, difficulty to attend the programmed visits or refusal to participate in the study.

### Randomisation

Patients will be randomly assigned to either penicillin V 1,600,000 units (two 800,000- unit pills) or amoxicillin 1,000 mg (two 500-mg pills) both taken thrice daily during 10 days. Subject numbers will be assigned sequentially as each subject enters the study. The subjects will be assigned a study drug through a randomisation schedule based on the randomisation plan. Since this is a multicentre study a block procedure will be undertaken for the assignment of each primary care centre participating in the study. Each container will include 66 pills (60 pills for the treatment plus 6 extra pills). The study drug, which will be prepared by the Pharmacy Unit of Hospital Universitari Son Espases (Palma de Mallorca, Spain) and will be labelled with the study number and a unique identification number.

Use of antithermic drugs or analgesics (acetaminophen, acetylsalicylic acid or ibuprofen), bronchodilators as well as any medication, except oral systemic antibiotics, that the patient is taking and which have been initiated prior to inclusion in the study will be allowed. Since this is a clinical study no statistically significant differences are to be expected in the concomitant treatment administered in the two treatment arms. In the case of clinical deterioration, the investigating physician will administer another antibiotic treatment in accordance with the clinical criteria.

### Blinding issues

We will perform a double-blind clinical study in which neither the physician nor the patient will know the treatment administered. The radiologists responsible for diagnosing pneumonia and confirming the radiological cure will be also blind to the clinical data and the treatment administered. The two medications will be presented as the same type of capsule. This will ensure that even on opening the container no one will know which medication has been given.

### Withdrawal

Each participant will have the right to withdraw from the study at any time. In addition, the investigator may discontinue a participant from the study at any time if the investigator considers it necessary for any reason including significant protocol deviation, significant non-compliance with the treatment regimen or study requirements (the patient will be required to return all the medication samples not taken to the investigator; if the remaining medication is not returned, compliance will be evaluated as insufficient), an adverse event which requires discontinuation of the study medication or results in inability to continue to comply with study procedures, disease progression which requires discontinuation of the study medication or results in inability to continue to comply with study procedures. In cases of clinical deterioration the medical investigator will implement another antibiotic treatment considering the clinical criteria, withdrawal of consent or loss to follow up.

### Outcome measures

The variable of the main result will be clinical cure at 14 days, defined as absence of fever, resolution or improvement of cough, improvement of general wellbeing and normal respiratory auscultation or reduction of crackles indicating that no other antimicrobial treatment is necessary. Any clinical result other than the anterior will be considered as treatment failure. Variables of secondary results will be efficacy at day 30 after the initiation of antibiotic treatment, radiological resolution or improvement one month after the initiation of the treatment, complete clinical resolution at day 14 defined as the total resolution of acute symptoms and signs related to the infection, the presence of adverse events which may appear during the treatment, and compliance with treatment administered. The radiologists responsible for diagnosing pneumonia and confirming the radiological cure will be blind to the clinical data and the treatment administered.

### Ascertainment of visits

On fulfilling the inclusion criteria the nature of the study will be explained to the patient and informed consent will be requested. All the eligible patients should have a chest X-ray (posteroanterior and lateral projection) demonstrating pneumonic infection. The study scheme and the visit program will be explained to the patient (Table 1). The patients will be randomised to one of the 2 treatment groups and the medication given. The first dose of the drug will be administered in the presence of the investigator. They will be dispensed the drugs for ten days.

**Table 1:** Variables to be collected during the clinical trial

	**Visit 1**	**Visit 2**	**Visit 3**	**Visit 4**
	**Day 0**	**Day 3**	**Day 14**	**Day 30**
**Medical history and examination**	x	x	x	x
**Pulse oximeter determination**	x	x	x	x
**Chest X-ray**	x			x
**Informed consent form**	x			
**Randomisation**	x			
**Evaluation of clinical evolution**		x	x	x
**Assessment of adverse events**		x	x	x
**Review of compliance**		x	x	

At the first follow-up visit, scheduled at day 3, a worsening of the clinical situation of the patient will be evaluated to determine whether a change in the antibiotic treatment is necessary. Likewise, compliance and possible secondary effects of the treatment will be evaluated. At the second follow-up visit, at day 14, the clinical evolution of the signs and symptoms will be evaluated and the need to change the antibiotic treatment in the case of worsening in the clinical manifestations will be determined. Likewise, compliance and possible secondary effects of the treatment will be evaluated. The last follow-up visit, scheduled at day 30, the clinical outcome of the signs and symptoms of the pneumonia will be evaluated and a new chest X-ray will be performed (posteroanterior and lateral projection) to confirm radiological resolution of the pneumonia. The medication will be discontinued in the case of clinical failure in follow-up visits 2 and 3 or in the case of significant adverse events.

The variables will be registered in an electronic case report form designed by the Hospital Universitari Vall d’Hebron (Barcelona, Spain). The variables considered in this trial will be sociodemographic (birth date, gender, race) and toxic habits (smoking and drinking behaviours), present medical history (presence and duration of signs and symptoms: fever, cough, expectoration, sputum purulence, diarrhoea, malaise, muscular aches, joint aches, thoracic pain, dyspnoea, respiratory auscultation; and details of any history of high blood pressure, type 2 diabetes mellitus or hypercholesterolemia), physical examination (axillary temperature, resting pulse, respiratory rate, blood pressure measurements, oxygen saturation, and auscultation abnormalities) and X-ray findings.

### Sample size

The objective of the study is to demonstrate that penicillin V is not inferior to amoxicillin. Considering a success rate of 85% for the group treated with amoxicillin [20,21] a total of 105 patients will be required in each treatment group (total of 210) to detect a non-inferiority margin of 15% at maximum between the two treatments with a minimum power of 80% considering an alpha error of 2.5% for a unilateral hypothesis and maximum possible losses of 15%.

### Statistical analyses

The intention-to-treat (ITT) population will include all randomised patients receiving at least one dose of study drug and the per-protocol (PP) population will include patients receiving no systemic antimicrobial agents other than the study drug for at least three days in the case of clinical failure or ≥80% of study medication in the case of cure, with adequate assessment of compliance and absence of major protocol violations.

To evaluate the comparability of the groups the two groups will be analysed with variables expressed as means and standard deviations for the case of quantitative variables and with proportions in the case of qualitative variables. The variable of the principle result, clinical cure, will be expressed as percentages and the comparison of percentages in the two treatment groups will be analysed using the Chi-square test. Logistic regression will be performed for the analysis of the predictive factors of cure or not, with calculation of the odds ratio for each of the variables analysed and multiadjustment for each of the factors of the study with confidence intervals of 95%. Variables with a p<0.20 on bivariant analysis will be included in the analysis. A p value < 0.05 will be considered statistically significant.

### Limitations and strengths

The main limitation of this study is that the microbiological study is not taken into account. This study will be carried out within the primary care setting in which 80% of community-acquired pneumonias are treated. Bacterial eradication has been considered a secondary outcome in many studies undertaken up to now. In a recent review published by Gilbert this measure was not recommended in mild-moderate pneumonia [22]. Despite the use of aggressive tests such as serological analysis, sputum samples, blood cultures, etc., to identify the causal agent of pneumonia, the causative microorganism of the pneumonia is only identified in 20% of ambulatory cases. Moreover, primary care physicians judge the response to treatment with eminently clinical but not with microbiological criteria. Neither is the return to daily activities of the patients considered because of the coexistence of variables which cannot be controlled and which cannot be generalised to other countries. The percentage of referral to hospital will not be taken into account since many other confounding variables lay behind this variable in a study of these characteristics. Neither will blood tests be carried out. Although they may feasibly be done within the primary care setting, they are not justified in this study since this is not a study on safety.

This study will be undertaken in several health care centres with an investigator-monitor figure in each. Previous meetings with the investigators and all the participating physicians of the different health care centres will allow the homogeneity necessary to carry out the study.

Because penicillin is seldom used in our country and because such a high dose of penicillin is even less prescribed, a Data and Safety Monitoring Board will be created, constituting a strength of the study. An independent group of experts will be responsible for monitoring patient safety and treatment efficacy data during recruitment. From a microbiological standpoint, bacterial eradication is achieved when the concentration of antibiotic is above 40-50% of the time between β-lactams dose intervals. For partially resistant strains, it is recommended that this time be above 60% [23]. We know that the bioavailability of penicillin V is very variable, ranging from 25% and 60%. The concentration of penicillin measured 1–3 hours after the last consumption of a 250-mg penicillin V regimen taken four times daily for 3–4 days is 0.1 - 2 μg/ml [23]. One study found that the MIC achieved with penicillin V is higher than with penicillin G administered parenterally [24]. In a study carried out in Sweden, Fredlund *et al.* showed that oral penicillin V is as effective as parenteral penicillin in patients with community-acquired pneumonia when the former is administered during ten days [25]. In the Scandinavian countries, where penicillin V is considered as the first choice for uncomplicated pneumonia [1], high doses ranging from 1 million to 1.5 million units thrice-daily are recommended [26,27]. The dose of 1.6 million units has been chosen in our study, since the percentage of intermediate pneumococcal resistance strains is much greater in Spain than in the Scandinavian countries. According to data from the European Centre for Disease Prevention and Control for the year 2011, intermediate pneumococcal resistance to penicillin was 20.4% in Spain, 4.6% in Denmark, 3.4% in Norway and 0.3% in Sweden [28]. Regarding the safety of the regimen of 1.6 million units (corresponding approximately to 1 gram of penicillin V) thrice-daily, although this dose is not marketed in our country, a presentation of 1.5 million units is available in Scandinavian countries. In addition, the British National Formulary recommends doses of up to 1 gram of penicillin V every 6 hours [29]. For this reason, we think that the dose of 1.6 million units taken thrice daily is fully justified in this clinical trial.

### Ethical aspects

The study will be conducted in accordance with the principles of the Declaration of Helsinki, ICH Guidelines for GCP and in full conformity with prevailing regulations. The study has been approved by the Ethical Committee of Investigation in Primary Care in Catalonia (*Fundació d’Investigació en Atenció Primària*) (protocol code: IJP–PEN-2012) (Additional file 1) and by the *Agencia Española del Medicamento y Productos Sanitarios*. This study does not require the use of unusual, additional tests: only a chest X-ray will be undertaken to rule out a pneumonic process, being a test that is total justified in this project. From an ethical point of view, this is to certify that the objective of this study is important and relevant for primary care, the power of the study may be considered as reasonable, this is an original study, the risks which the participants may incur justify the investigation being carried out with a totally favourable benefit/risk quotient, and we ensure the external validity of the study to the primary care reality, with the inclusion and exclusion criteria described. Vulnerable populations will not be participating in this study and neither will economic compensation be given to patients for their participation. The investigators will be free to publish the results of this study, regardless of the results obtained.

The trial staff will ensure that the anonymity of the participants is maintained. The participants will be identified only by a participant number on the consent report form and an electronic database. All documents will be stored securely and only accessible by trial staff and authorized personnel. The study will comply with the Data Protection Legislation which requires data to be anonymised as soon as it is mandatory to do so.

## Discussion

In this study, interventions and follow-ups will be similar to clinical practice. We believe that the methodology used is very simple as it should be in primary care. The data collection has been simplified to facilitate the inclusion of cases in primary care offices. Chest x-ray is absolutely necessary to confirm the diagnosis of pneumonia and will therefore be an essential requisite for inclusion of the patients to thereby avoid the inclusion of patients who do not have the disease. Secondly, this study will be undertaken in several health care centres with an investigator-monitor figure in each. The antibiotics used in this study have been in the Spanish pharmaceutical market for more than 30 years, being amoxicillin the first-drug of choice in our guidelines for the treatment of pneumonia in the community setting in adults under 65 years [12,13]. Therefore, the principle of equipoise is guaranteed in this clinical trial. Since the dose of penicillin considered in this trial is high and infrequently used in our country, a Safety Board will be the responsible body to monitor the adverse events that might be present during the development of the trial, thereby ensuring the safety of the patients recruited. Only uncomplicated cases of pneumonia that could be treated in the community will be taken into account. Even though patients with certain comorbidities such as high blood pressure or type 2 diabetes mellitus might be included in this trial we believe that the sample size and the randomised, double-blind methodology used in this study will eliminate a possible selection bias.

This study may have a socioeconomical impact. If treatment with penicillin demonstrates to be as effective as that of amoxicillin in uncomplicated community-acquired pneumonia, this will have a considerable socioeconomic impact. It may also have an impact on the treatment of other respiratory tract infections. Most prescriptions of amoxicillin and amoxicillin-clavulanate are prescribed for respiratory tract infections [3]. If treatment of the most severe respiratory infection (pneumonia) with penicillin is found to be as effective as that with a broad-spectrum β-lactam, this would aid in adopting a more rational use of antibiotics in respiratory infections mainly caused by pneumococci. Both the amount of antibiotics used and how they are used contribute to the development of resistance. The use of broad-spectrum antibiotics rather than narrow-spectrum drugs is known to favour the emergence of resistance by broadly eliminating competing susceptible flora [30]. If the efficacy of penicillin is comparable to the first-choice drug for this infection we will be able to encourage the use of narrower-spectrum antibiotics for treating pneumococcal respiratory tract infections. On the other hand, penicillin V is less expensive in our country and this would therefore also aid in reducing drug costs for the treatment of this infection.

## Competing interests

The authors declared that they have no competing interest.

## Authors’ contributions

CL and JA conceived the study. CL, JA, RM, AGS, JL and MGS contributed to the planning of the study. CL was responsible for drafting the paper. All authors revised and approved the final version of the manuscript.

## Pre-publication history

The pre-publication history for this paper can be accessed here:

http://www.biomedcentral.com/1471-2296/14/50/prepub

## Supplementary Material

Additional file 1 Research Ethics Committee Report. Click here for file
