# Supplementary material for: Efficacy of high doses of oral penicillin versus amoxicillin in the treatment of adults with non-severe pneumonia attended in the community: study protocol for a randomised controlled trial
Source: BMC Fam Pract. 2013 Apr 17;14:50. doi: 10.1186/1471-2296-14-50 (PMC3637575; doi:10.1186/1471-2296-14-50)
Supplement: Additional file 1 — Research Ethics Committee Report. [file 1471-2296-14-50-S1.pdf]

RESULTADO EXPEDIENTE - PI11/02471

Centro Solicitante: IDIAP JORDI GOL

Centro Realizador: CAP JAUME I

Título: Eficacia de dosis altas de penicilina V oral frente a amoxicilina a dosis altas en el tratamiento de la neumonía no grave atendida en la comunidad en adultos

RESOLUCIÓN PROVISIONAL DE CONCESIÓN

Estado de Resolución Provisional de Concesión : CONCEDIDO

PRESUPUESTO CONCEDIDO PROVISIONAL				
	1ª ANUALIDAD	2ª ANUALIDAD	3ª ANUALIDAD	TOTAL
BIENES/SRV	42.341,00	28.227,00	23.523,00	**94.091,00**
PERSONAL	0,00	0,00	0,00	**0,00**
VIAJES	1.500,00	750,00	750,00	**3.000,00**
**SUBTOTALES**	**43.841,00**	**28.977,00**	**24.273,00**	**97.091,00**
Costes ind. 21,00 %	9.206,61	6.085,17	5.097,33	20.389,11
**TOTALES**	**53.047,61**	**35.062,17**	**29.370,33**	**117.480,11**

PERSONAL CONCEDIDO PROVISIONAL CON CARGO AL PROYECTO

Personal con Cargo	Concedido Provisional
Titulado superior	0
Titulado medio	0
Técnico FP	0

EQUIPO DE INVESTIGACIÓN

Nombre	Apellido 1	Apellido 2	Tipo	Ded.
MARC	MIRAVITLLES	FERNANDEZ	Colaborador	COMPARTIDA
JAVIER	ARRANZ	IZQUIERDO	Colaborador	COMPARTIDA
SILVIA	HERNANDEZ	ANADON	Colaborador	COMPARTIDA
JORDI	BLADE	CREIXENTI	Colaborador	COMPARTIDA
CARLES	LLOR	VILA	IP	UNICA
EUGENIA	CARANDELL	JAGER	Colaborador	COMPARTIDA
JESUS	ORTEGA	MARTINEZ	Colaborador	COMPARTIDA

RESOLUCIÓN DEFINITIVA DE CONCESIÓN

Estado de Resolución Definitiva de Concesión : CONCEDIDO

PRESUPUESTO CONCEDIDO DEFINITIVO				
	1ª ANUALIDAD	2ª ANUALIDAD	3ª ANUALIDAD	TOTAL
BIENES/SRV	42.341,00	28.227,00	23.523,00	**94.091,00**
PERSONAL	0,00	0,00	0,00	**0,00**
VIAJES	1.500,00	750,00	750,00	**3.000,00**
**SUBTOTALES**	**43.841,00**	**28.977,00**	**24.273,00**	**97.091,00**
Costes ind. 21,00 %	9.206,61	6.085,17	5.097,33	20.389,11
**TOTALES**	**53.047,61**	**35.062,17**	**29.370,33**	**117.480,11**

PERSONAL CONCEDIDO DEFINITIVO CON CARGO AL PROYECTO

Personal con Cargo	Concedido Definitivo
Titulado superior	0
Titulado medio	0
Técnico FP	0

EQUIPO DE INVESTIGACIÓN

Nombre	Apellido 1	Apellido 2	Tipo	Ded.
MARC	MIRAVITLLES	FERNANDEZ	Colaborador	COMPARTIDA
JAVIER	ARRANZ	IZQUIERDO	Colaborador	COMPARTIDA
SILVIA	HERNANDEZ	ANADON	Colaborador	COMPARTIDA
JORDI	BLADE	CREIXENTI	Colaborador	COMPARTIDA
CARLES	LLOR	VILA	IP	UNICA
EUGENIA	CARANDELL	JAGER	Colaborador	COMPARTIDA
JESUS	ORTEGA	MARTINEZ	Colaborador	COMPARTIDA
